# The Effects of Suspension Training on Dynamic, Static Balance, and Stability: An Interventional Study

**DOI:** 10.3390/medicina60010047

**Published:** 2023-12-26

**Authors:** José-María Blasco, Fernando Domínguez-Navarro, Catalina Tolsada-Velasco, Irene de-Borja-Fuentes, Elena Costa-Moreno, Carmen García-Gomáriz, María-José Chiva-Miralles, Sergio Roig-Casasús, David Hernández-Guillen

**Affiliations:** 1 Group in Physiotherapy of the Ageing Process: Social and Healthcare Strategies, Departament de Fisioteràpia, Universitat de València, 46010 Valencia, Spain; jose.maria.blasco@uv.es (J.-M.B.); sergio.roig@uv.es (S.R.-C.); david.hernandez@uv.es (D.H.-G.); 2 Departament de Fisioterapia, Facultat de Fisioteràpia, Universitat de València, 46010 Valencia, Spain; catalina.tolsada@uv.es (C.T.-V.); irene.borja@uv.es (I.d.-B.-F.); elena.costa@uv.es (E.C.-M.); 3 IRIMED Joint Research Unit (IIS-LaFe-UV), 46010 Valencia, Spain; 4 Facultad de Ciencias de la Salud, Universidad Europea de Valencia, 46010 Valencia, Spain; 5 Departament d’Infermeria i Podologia, Universitat de València, 46021 Valencia, Spain; carmen.garcia-gomariz@uv.es (C.G.-G.); maria.jose.chiva@uv.es (M.-J.C.-M.); 6 Hospital Universitari i Politècnic La Fe de València, 46026 Valencia, Spain

**Keywords:** suspension training, balance, functionality, posturography

## Abstract

*Background and Objectives:* While suspension training devices are increasingly gaining popularity, there is limited evidence on their effects on balance, and no comprehensive assessment has been conducted. This study aimed to evaluate the effects of a 9-session suspension training program on dynamic and static balance, stability, and functional performance. *Materials and Methods:* A total of forty-eight healthy adults, aged between 18 and 30, participated in a 9-session suspension training program. The program included exercises targeting upper and lower body muscles as well as core muscles. Balance was comprehensively assessed using various dynamic balance tests, including the Y Balance Test (YBT) as the primary outcome, single-leg Emery test, and sideways jumping test. Static balance was evaluated through the monopedal and bipedal Romberg tests. Changes from baseline were analyzed using a one-way ANOVA test. *Results*: Thirty-nine participants (mean age: 21.8 years) completed the intervention. The intervention resulted in significant improvements in YBT, jumping sideways, Emery, and 30s-SST scores (*p* < 0.001). Platform measures indicated enhanced monopedal stability (*p* < 0.001) but did not show a significant effect on bipedal stability (*p* > 0.05). *Conclusions*: Suspension training is a safe and feasible method for improving dynamic balance and functional performance in healthy, untrained young adults. However, it does not appear to significantly impact the ability to maintain a static posture while standing.

## 1. Introduction

Balance is a critical physical capacity essential for daily activities and sports development. It can be defined as the coordination of movement strategies to stabilize the center of mass during both static and dynamic conditions. However, as highlighted by Horak et al. [1], the balance may not be considered merely as the summation of static reflexes, but a complex ability based on the interaction of dynamic sensorimotor processes. Achieving adequate postural control, inherent in the efficient ability to stand, walk, and interact with the environment safely and efficiently, demands the involvement of various physiological systems and responses. These encompass sensory and movement strategies, spatial orientation, and control of dynamics [2].

Training and restoring balance involve a multimodal approach, with task-performance exercises oriented to activate core and lower-limb muscles, stimulate proprioception [3,4], and challenge stability by modifying base of support [5]. Different devices can be used to achieve this purpose, with suspension training devices becoming increasingly more popular in the sports and clinics fields over the last decade [6]. Suspension training involves working with resistance that is exerted by the own body weight of the individual. The load can be modulated with the degree of inclination adopted [7], which leads to physiological positive effects in terms of muscle activation and strengthening [8,9]. Furthermore, it creates a continuous state of instability, which solicitates postural correction strategies and induces a high level of neuromuscular stimulation, which may enhance balance capacities [10].

Suspension devices have been employed in various studies for stability-based strength training programs, encompassing heterogeneous groups of individuals. These groups comprise athletes [11], older adults [9], individuals sport injuries [12], and those with orthopedics and neurological conditions [13,14]. Within these studies, suspension devices are used to perform core, lower-, and upper-limb activation exercises, executed with different degree of suspension depending on the subject ability. These exercises encompasses training programs, lasting from 8 to 24 sessions, that can be executed either in isolation or integrated into conventional strength training programs or standard care [8,9,10,11,13].

The assessment of the effects of suspension training has traditionally focused on strength and muscle properties, with limited evidence on balance components. Among the studies evaluating balance components, some observed benefits after a training program for dynamic balance, measured with performance-based tests [9,15] and dynamometric platforms [12], while others did not observe such improvements [11,14]. Also, contradicting results are found for functional outcomes [9,13]. Consequently, current evidence on the effects of balance and functional capacity is still limited, with few studies assessing these effects, provided from a heterogeneous population, and reporting non-uniform results. Furthermore, these studies have focused solely on the specific balance parameters, neglecting the intricate system interaction involved in balance. Therefore, a comprehensive analysis for the balance effects is still missing. To achieve this purpose, different balance dimensions (static balance, dynamic balance, and stability), combining both performance-based and technological equipment measurements, should be performed.

Dynamic balance can be measured using performance tests such as the Y Balance Test (YBT) [16] or Timed Up and Go (TUG) [9], which have proven validity in assessing the ability to effectively displace the center of mass in young and older adults, respectively. Moreover, balance assessment covers the evaluation of both static scenarios, where no body movement is performed, and situations that do not involve shifts in body position or support. The Emery test [17] is specially designed for such purpose, allowing for compensation movements during evaluation. Center mass displacement might be assessed as well using technological equipment, such as dynamometric or stabilimeter platforms, to capture the swing area of the center of gravity (for static balance), and multiaxis mean trajectory difference (for dynamic balance) conditions, with excellent reliability exposed (ICC = 0.990) [18].

Furthermore, the incorporation of balance components into functional development can be evaluated through performance-based assessments like the 30 s Sit-to-Stand test or its equivalent, the timed 5 times Sit-to-Stand test. This is particularly relevant given the well-established direct correlation between balance and functionality [19] and the proven validity of these tests [20]. Such functional assessments provide a comprehensive understanding of an individual’s balance capabilities in real-world scenarios, contributing valuable insights to the overall evaluation of balance and its implications for daily activities.

By assessing a broader range of balance parameters, we can expect to gain more detailed knowledge of how suspension training may correct or enhance specific components of balance. This, in turn, could enable the development of more tailored interventions to address individual weaknesses or areas in need of improvement in both balance and functional capacity. For a preliminary understanding of these effects, it is logical to conduct assessments on healthy subjects. This allows for a pure understanding without the interference of factors with heterogeneous interactions and implications, such as pain, neuro-muscular alterations, and diminished proprioception, which are common in neurological and orthopedic conditions [21,22]. Therefore, the objectives for the present study are: (I) to evaluate the effects of suspension training for static balance, (II) to assess the effects of suspension training for dynamic balance, (III) to evaluate posturography stability (IV), and to measure the effects on performance-based functionality. Consequently, the hypothesis for the study is that a 9-session suspension training program will be a feasible and effective training to improve the balance in healthy untrained young adults. 

## 2. Materials and Methods

This was a quasi-experimental study conducted at the Universitat de València (Valencia, Spain), between March and June 2019, and written in accordance with the CONSORT statement. The study was prospectively registered in ClinicalTrials.gov with the identifier NCT03889665 and received approval from the Human Research Ethics Committee of the Universitat de València (H1549898795086). All participants were informed about the methods and gave written consent to participate. The procedures adhered to the principles of the Declaration of Helsinki. The research included 48 healthy, untrained, young adults, aged between 18 and 30 years old, who volunteered to participate. Exclusion criteria were: (I) professional athletes, (II) musculoskeletal injury in the last 6 months (e.g., sprain), and (III) known balance disorders such as vertigo, central or vestibular affection.

### 2.1. Intervention

The participants underwent a supervised suspension training program of 9 sessions, using the commercial TRX^®^ (San Francisco, CA, USA) suspension equipment. All sessions were implemented in the same facilities and supervised by the same researcher. The program was designed for full-body training, incorporating exercises for the upper and lower body, as well as core exercises. The program’s structure and included exercises were based on previously published articles [9] and the research team’s own experience with training using this device.

Each session began with a 5–10 min warm-up, encompassing mobility exercises for both upper and lower limbs, along with the execution of one upper-limb and one lower-limb exercise using the TRX with minimal load. Subsequently, participants followed the same two-block structure in each session. The first block consisted of 6 upper and 6 lower body exercises that were performed alternatively. Exercises for upper body included:

- Low row: By pulling the handles towards the body at a lower angle, participants primarily target the muscles of the upper back, including the latissimus dorsi.
- High row: In contrast to the low row, the high row focuses on the upper fibers of the back muscles. Participants pull the handles downward, activating the rear deltoids and rhomboids.
- Biceps curl: This exercise isolates and strengthens the biceps brachii muscles. By adjusting body angle and resistance, participants can tailor the intensity of the exercise to their fitness level.
- Y-fly: Participants, suspended at an angle, perform a controlled outward movement resembling the letter ‘Y’. This exercise enhances engaged muscles of the shoulder and upper back.
- Elbow extensions: Participants, by extending their arms against the resistance, effectively isolate triceps brachialis.
- Chest press: This exercise involves pushing the body away from the anchor point, targeting the pectoral muscles.
- Lower-body exercise program was compound by:
- Double-leg squat: It consists of engaging both legs simultaneously by performing a sitting movement, while holding the suspension device.
- Step-front right lunge: Participants performed a stepped forward with the right leg, while holding the suspension device, emphasizing quadriceps, hamstrings, and glutes activation. Furthermore, it creates an anteriorization of the center of gravity.
- Step-front left lunge: With the same indication to the previous exercise but performing the stepped forward with left leg.
- Sideway lunge: Similarly, while participants maintain tension on the suspension device, perform a lateral step alternatively with each leg. The center of gravity is displaced laterally.
- Step-back right lunge: By this exercise, the right leg is stepped backward, while tension is maintained on the suspension device and the center of gravity is displaced posteriorly.
- Step-back left lunge: Same indication performance as the previous exercise, but with left leg stepping.
- The second block consisted of 6 exercises that targeted core musculature:
- Pike: Participants suspended themselves at an angle and performed a controlled hip movement towards the anchor point, resembling a pike position.
- Mountain climbers: This exercise involved participants bringing their knees towards the chest in a dynamic fashion while suspended.
- Frontal plank: individuals maintained a static position by supporting their body with the suspension device, facing the floor.
- Side right plank: This exercise was executed by supporting the body on one arm while suspended, similarly to the frontal plank, bur rotating the whole 90 degrees to the right.
- Side left plank: With the same indications as the previous one, but with the body oriented 90 degrees to the left.
- Glutes bridge: It involved participants lifting their hips towards the anchor point while suspended.

There was a 3 min rest between each block, while the sessions lasted from 25 to 30 min. The training dose increased every three sessions by adding 2–3 more repetitions per set, or increasing the time duration of exercise, as explained in detail in Appendix A. The training intensity was aimed to be moderate–high, corresponding to a rate of perceived exertion of a rating score of 7–9 on a scale of 0 to 10. Each participant adjusted the body suspension using the TRX to achieve this intensity. The description of the exercises and the details of the intervention are shown in the Appendixes (Appendix B), along with images of the performed exercises (Appendix C, Appendix D and Appendix E).

Two sessions per week were carried out, with a minimum rest of two days between sessions. In this trial, we considered that the program was completed if a participant attended at least 7 sessions (80% compliance). The data of participants with lower compliance rates were not analyzed. Participants were advised to report any physical complaints potentially derived from the training.

### 2.2. Outcomes and Prioritization

By the proposed objectives, the present study comprehensively evaluated balance, including tests related to static and dynamic balance, and stability, following the previously published classifications and recommendations on the assessment of balance [1,23]. Moreover, performance-based functionality was also evaluated. Outcomes were prioritized as follows:

#### 2.2.1. Primary Balance Outcome

- Y Balance Test: This test is used to evaluate dynamic balance in adults and athletes, which requires strength, proprioception, and flexibility of the lower limbs. The YBT is the simplified version of the original Star Excursion Balance test, in which balance capacity when moving in 3 of the 8 directions (i.e., anterior, posteromedial, and posterolateral) is measured. This test has reported excellent inter- and intra-rater reliability (ICC of 0.88 and 0.90, respectively) in healthy populations [16]. For the present study, the test was performed with the Y-Balance instrument kit, a device consisting of a stance platform to which three pieces of PVC pipe are attached to the three directions of movement; the reliability of this equipment has been evidenced [24]. As for the test, participants were instructed to stand on one foot and, with the contralateral limb, push the pipe along the line direction, reaching as far as possible, without compromising their balance. The participants were barefoot, and the hands had to remain on the hips. The reached distance was recorded in centimeters, and the total score was calculated for each one of the 3 directions and standing on each limb. The results were expressed about the leg length, according to the following formula: summative score/3 * length leg [25].

#### 2.2.2. Secondary Balance Outcomes

- Single-leg Emery test: This is a timed balance test that was specifically designed to assess the static and dynamic balance of young people and adolescents [17]. The Participants performed the test barefooted and with closed eyes and were asked to stand on one leg on an Airex^®^ Balance-Pad, with the weight-bearing leg with slight knee flexion, and the non-weight-bearing leg with approximately 45 degrees of flexion. Hands had to be placed on the hips. It was recorded the time that the participant could maintain the single-leg position without touching the floor or the weight-bearing limb with the non-weight-bearing limb, opening the eyes, or removing the hands from the hips.
- Jumping sideways test: This is a timed test that assesses motor coordination and dynamic balance under time pressure [26]. The participants were required to jump sideways keeping their feet together while jumping laterally at a distance of 60 cm as fast as possible for 15 s. The score was obtained from the total number of correct jumps. A jump was considered incorrect if the feet were separated during the jump or if the jump distance was less than the established.
- Static stability/standing balance: A T-Plate^®^ platform was used to assess static standing balance and stability while keeping a static upright position. This allowed us to assess the functioning of sensory pathways, sensorimotor integration, and motor pathways. The participant was instructed to stand keeping a static upright position on both feet, with slight knee flexion, and the eyes opened for 30 s. The excursion of the center of pressure [27] was measured in terms of the swayed area (mm^2^) and velocity (m/s).
- Monopedal stability: The single-leg stand test, a timed test that assesses the capacity to keep balance on one limb, was measured on a T-Plate^®^ platform. The participants were allowed to decide the limb to stand on for 30 s, and the choice was recorded to repeat the procedure in the post-intervention assessment. The excursion of the center of pressure in both tests was then estimated in terms of the swayed area (mm^2^) and velocity (m/s).
- The 30 s sit-to-stand test (30s-SST) [28] is a test designed for testing leg strength and endurance and was used to assess balance and functional performance in this study. To perform the test, a chair with a hard seat and backrest was used. Participants were instructed that they had to sit down and get up from the chair starting from a standing position with both feet flat on the floor. The arms had remained crossed on the chest with the hands touching the shoulders. The sit-to-stand procedure had to be performed as many times as possible for 30 s, the total number of sit-to-stand repetitions being considered as the total score on this test.

### 2.3. Measurement Procedures

First, a verbal explanation was given, and an attempt was allowed to become familiar with the tests. Then, the tests were performed twice, and the average score was used for further analysis. A minimum of 60 s rest between attempts, and of 3 to 5 min between tests, was allowed. The order to perform the tests was the same for all participants (as described above). Participants were assessed twice: firstly, three to 1 days before the intervention began, called pre-intervention assessment, and then, one to 2 days after the last training session, i.e., the post-intervention assessment.

### 2.4. Data Analysis

A descriptive analysis was used to present the demographic characteristics and clinical outcomes as means, standard deviations, and percentages when appropriate. The normal distribution of data was assessed using the Kolmogorov–Smirnov test. To evaluate the intervention time effects, a one-way analysis of variance for repeated measures was performed, comparing pre- and post-intervention results for each outcome. A significance level of *p* = 0.05 was set. These analyses were carried out with SPSS version 22 software.

## 3. Results

Overall, 39 participants (82% of the included sample) completed the training program. All the drop-out cases were related to non-compliance with the required attendance rate (<80% of attendance). No adverse effects were reported. Table 1 shows the demographic characteristics of the participants and the average attendance rate.

After the 9-session program, different balance components were enhanced. It was observed a significant improvement in YBT scores (*p* < 0.001), with a mean increase in the reached distance of 4.39 cm and 4.34 cm at each limb. Moreover, significant basal changes were observed in the Emery (right leg, *p* = 0.001; left leg, *p* < 0.001) and jumping sideways tests (*p* < 0.001). The participants also improved the measured monopedal velocity (*p* < 0.001), but there were no improvements in the posturography measures of sway area and velocity (*p* > 0.05). Finally, the 30s-SST scores significantly improved after the training program. The details of the results are presented in Table 2.

## 4. Discussion

This study hypothesized that a 9-session suspension training program will be feasible and effective to enhance balance in healthy untrained young adults. The obtained findings mostly support the hypothesized effectiveness, revealing significant balance improvements after the training program, especially in terms of dynamic balance, with a moderate-to-large effect size observed. Moreover, the balance enhancement might be transferred to functional performance since values from 30s-SST significantly improved after the 9-session training. However, inconsistent improved outcomes were found in stability measurements. Moreover, no adverse effects were recorded in the analyzed participants; therefore, it confirms that, among healthy untrained young participants, suspension training is safe and feasible. To the best of our knowledge, this is the first study in describing comprehensively the effects of suspension training in balance, assessing a wide range of outcomes that covers the different domains of balance: static, dynamic, posturography stability and functional performance. With the obtained findings, it is corroborated that training with suspension devices may enhance balance components, as suggested in other studies reporting improvements in static [12] and dynamic balance [9,14], while the observed improvements in functional performance constrain with the lack of effect on task domain found in other studies [13].

A suspension training device, such as commercial TRX^®^, provides the opportunity to work with different loads and weight transfers, creating an unstable environment that challenges balance systems [29] and enhancing core and lower-limb muscles activation [30]. This training scenario may lead to improved different dimensions of balance. In addition, training with a suspension device requires coordinating the distribution of loads between the upper and lower body, which places a greater demand on the core musculature [31], which has been previously reported to improve muscle activation and strength of these muscles [8,30]. Those neuromuscular adaptations may serve as an explanation, particularly for dynamic balance outcomes, where the demands are more significant.

Concretely, the enhanced dynamic balance was observed through different tests: YBT, jumping sideways and Emery test. Overall, our research found basal improvements in the YBT above 4 cm. This range of improvement is comparable to that obtained by other studies when performing similar exercise programs, as in the case of Benis et al. [10], who carried out weight-bearing neuromuscular training in healthy basketball players, observing a range of 3–4 cm improvements depending on the tested direction; as well as in the study by Rapelt et al. [32], where healthy young adults implemented a short interval training program, showing a 3.37 cm improvement after training. Benefits in this area can be attributed to whole-body muscle activation exerted during suspension training, necessary for stabilizing and coordinating different body segments during dynamic tasks [33]. Likewise, it should be taken into account that YBT results should be understood in the context of altered motor control and limited ankle mobility potentially influencing the obtained outcomes [34].

The observed results align with prior studies suggesting positive effects on dynamic balance outcomes associated with suspension training. Huang et al. [12] conducted an assessment utilizing posturography to evaluate the impact of a 6-week program on subjects rehabilitating from anterior cruciate ligament injury. Their results revealed a significant reduction in the average trace error, from 33.05 (SEM: 8.64) to 24.79% (4.88), indicative of enhanced dynamic balance capacity.

In the context of older adults, the TUG test serves as a commonly employed measure of dynamic balance, with a shorter test duration reflecting superior balance capacity. Soligon et al. [9] reported a noteworthy decrease in TUG test time (from 7.46 s (0.72) to 6.80 s (0.56)) following a 24-session suspension training program. However, the suspension training improvements were not superior to those achieved through a conventional exercise program. Similarly, Jimenez-Garcia et al. [22] also reported a significant pre-after training program differences, with a substantial effect size (d = 1.67) in the test, by implementing high-intensity training with suspension devices among older adults. However, Soligon et al. [9] found that this improvement was not superior to the one obtained by a conventional exercise program. Additionally, the Functional Reach Test, which is also employed for dynamic balance in older people, exhibited significant improved outcomes with a large effect size (d = 1.15) after a 6-week training program, according to the study conducted by Pierle et al. [15]. In summary, it is suggested that dynamic balance can be enhanced with suspension training not only in individuals with orthopedic injury or older adults but also on healthy young untrained subjects. However, considering the divergence of capacities between the different populations, functional demands, and the dosage for the training program should be appropriately adapted [35].

Despite observed enhancements among performance-balance tests, posturographic assessment did not reveal similar improvements. Bipedal stability did not exhibit significant enhancements; however, intriguingly, conflicting results were noted in monopedal stability. The swayed area demonstrated worse outcomes following training, yet there was a significant enhancement in velocity. The observed results constrain with other studies, such as those conducted by Huang et al. [12] and Yalfani et al. [14], where improvements in monopedal stability were identified. However, it is crucial to note that the participants in these studies had anterior cruciate ligament and abdominal diastasis injuries, respectively, conditions known to compromise balance capacity. Consequently, it can be hypothesized that among healthy adults, the observation of stability enhancements is more challenging due to higher capacities. Furthermore, it is plausible that even acute or delayed fatigue post-training may exacerbate the outcomes related to monopedal stability.

In terms of postulating explanations for the observed divergent balance effects between dynamic balance and stability, we may draw upon Horak’s [1] conceptual framework, which delineates balance into two dimensions: orientation in space and control of dynamics. While the first dimension depends more on vestibular, visual, and proprioceptive systems, the second is more related to the neuromuscular strategies oriented to stabilize the body’s center of mass [36]. Assuming so, it seems that suspension training is more focused on the enhancement of the latter and may explain why benefits seem to be greater in dynamic than static balance.

Simultaneously, functional performance appears to be enhanced by suspension training, as evidenced by an observed increase in the average count of three repetitions in the 30s-SST. These findings reinforce the idea that dynamic balance is a relevant element to functional performance [37], and benefits in one outcome are transferred to the other [38]. Moreover, as this test is strengthening- and resistance-demanding, improved muscle properties after suspension training reported in other studies may serve as well as an explanation [30]. In the literature, contradictory findings exist on the functional effects of suspension training. Inconsistent improvements were observed in the functional times sit-to-stand test by Soligon et al. [9], while in Emara’s study [13], no significant gains were observed in this test after an 18-session suspension training program among spastic diplegic cerebral palsy participants.

Suspension training is a relatively novel approach, and the evidence of its effects is not fully elucidated, primarily focusing on muscle activation and strength, but less on functionality and balance. This research contributes to extending the evidence of this training method by conducting a comprehensive assessment of various balance capacities. It provides a view of balance effects broader than that obtained for previous studies [6,22,39]. Furthermore, the evidence from these studies represents heterogeneous populations, with different ages of participants and conditions, which makes it challenging to standardize the expected balance effects derived from suspension training. From another perspective, these results cannot be directly extrapolated to individuals with underlying health conditions, given the pathophysiological impact that orthopedic, neurological, or geriatric conditions have on balance. However, the observed effects are robust, and consistently evident across various measures of balance.

In practical terms, the obtained results suggest that suspension training can be safely utilized to enhance balance, aligning with the positive effects observed in other instability devices [40]. The advantage lies in its ability to adjust intensity by modulating body weight, offering a versatile training approach. In addition, these findings provide a foundation for exploring suspension training as a potential tool for injury prevention, considering the well-established link between poor balance and an elevated risk of injury [38,41]. Additionally, after establishing the feasibility of enhancing balance through suspension training devices, it becomes pertinent to broaden the perspective by comparing these effects with those resulting from other training interventions, instability devices, or divergent training parameters.

The main strength of the present research lies in the comprehensive assessment of balance, combining both performance-based measures (YBT, jumping sideways, and Emery test) and objective measurements (posturography measures). Nevertheless, it is important to acknowledge some limitations. First, the results obtained were not compared to a passive control group or a group undergoing a different type of training, which may be a limitation to deeply understand its effects. Furthermore, the assessment of outcomes was confined to the short term, leaving uncertainty about the sustainability of observed benefits over longer periods. Lastly, the current literature lacks standardized protocols specifying the duration, exercises, and dosage suitable for suspension training. Consequently, the design of an evidence-based training protocol was not feasible within the scope of this study.

## 5. Conclusions

Suspension training is safe and feasible to enhance dynamic balance and functional performance in healthy untrained young adults but does not seem to have an impact on the capacity to maintain a static posture in an upright position.

## Figures and Tables

**Table 1:** Participant characteristics.

Sociodemographic Measure	Mean (SD)
Number of participants (n) Average attendance rate (%)	39 97.5
Age (years)	21.85 (3.49)
Women (n, %)	24 (61.5%)
Body mass index (kg/m^2^)	22.9 (3.3)

**Table 2:** Effects of interventions.

	Pre-Intervention	Post-Intervention			
Primary Outcome	Mean (SD)	Mean (SD)	*p*-Value *	Hedge’s Effect Size	Confidence Interval for Effect Size
YBT scores (average leg) (cm)	94.6 (8.5)	98.9 (6.6)	<0.001 *	0.565	−1.01 to −0.11
Right leg	94.5 (8.7)	98.9 (7.2)	<0.001 *	0.551	−1.00 to −0.09
Left leg	94.7 (8.8)	99.0 (6.3)	<0.001 *	0.568	−1.01 to −0,10
Secondary balance outcomes					
Emery test (s)					
Right leg	4.6 (2.8)	8.0 (7.1)	0.001 *	0.630	−1.08 to −0.17
Left leg	4.3 (2.2)	7.0 (3.9)	<0.001 *	0.852	−1.31 to −0.38
Jumping Sideways test (count)	29.5 (11.2)	34.2 (10.4)	<0.001 *	0.434	−0.88 to 0.02
Posturography measures					
Swayed area (mm^2^)	40.6 (47.3)	36.8 (30.0)	0.577	0.095	−0.35 to 0.54
Velocity (mm^2^/s)	2.1 (1.1)	2.0 (0.8)	0.412	0.104	−0.34 to 0.55
Monopedal swayed area (mm^2^)	292.2 (189.9)	368.6 (193.6)	<0.001 *	0.398	−0.84 to 0.05
Monopedal velocity (mm^2^/s)	13.1 (4.2)	15.0 (4.6)	<0.001 *	0.431	−0.88 to 0.02
Functional performance					
30s-SST (times)	17.0 (3.2)	20.2 (2.4)	<0.001 *	1.131	−1.60 to −0.64

*: statistically significant differences (*p* < 0.05); 30s-SST: thirty-second sit-to-stand test.

## Data Availability

Data will be available under reasonable request to the corresponding author.

## Appendix A

**Table A1:** Exercise Description and Dose Protocol for Suspension Training Program.

		Day 1 to 3	Day 4 to 6	Day 7 to 9
Upper body		Dose (sets × repetitions)		
UP-1	Low row	2 × 8	2 × 10	2 × 12
UP-2	High row	2 × 8	2 × 10	2 × 12
UP-3	Biceps curl	2 × 8	2 × 10	2 × 12
UP-4	Y-Fly	2 × 8	2 × 10	2 × 12
UP-5	Elbow extensions	2 × 8	2 × 10	2 × 12
UP-6	Chest press	2 × 8	2 × 10	2 × 12
Lower body		Dose (sets × repetitions)		
LB-1	Double-leg squat	2 × 5	2 × 8	2 × 10
LB-2	Step-front right lunge	2 × 5	2 × 8	2 × 10
LB-3	Step-front left lunge	2 × 5	2 × 8	2 × 10
LB-4	Sideway lunge	2 × 5	2 × 8	2 × 10
LB-5	Step-back right lunge	2 × 5	2 × 8	2 × 10
LB-6	Step-back left lunge	2 × 5	2 × 8	2 × 10
Core		Dose (sets × repetitions/time)		
C-1	Pike	2 × 5	2 × 8	2 × 10
C-2	Mountain climber	2 × 5	2 × 8	2 × 10
C-3	Frontal plank	2 × 15″	2 × 20″	2 × 30″
C-4	Side right plank	2 × 15″	2 × 20″	2 × 30″
C-5	Side left plank	2 × 15″	2 × 20″	2 × 30″
C-6	Glutes bridge	2 × 5	2 × 8	2 × 10

″: Means ‘seconds’.

## Appendix B

**Table A2:** Session Sequence.

Combined Upper- and Lower-Body Training Block (17–20)			
Order	Upper-body exercise	Lower-body exercise	Rest between exercise
1st	Low row	Double-leg squat	10″
2nd	High row	Step-front right lunge	10″
3rd	Biceps curl	Step-front left lunge	10″
4th	Y-Fly	Sideway lunge	10″
5th	Elbow extensions	Step-back right lunge	10″
6th	Chest press	Step-back left lunge	10″
3 min rest			
Core training block (8–10 min)			
1st	Pike		20″
2nd	Mountain climber		20″
3rd	Frontal plank		20″
4th	Side right plank		20″
5th	Side left plank		20″
6th	Glutes bridge		20″

″: Means ‘seconds’.
